# Cross-sectional association of dietary water intakes and sources, and adiposity: National Adult Nutrition Survey, the Republic of Ireland

**DOI:** 10.1007/s00394-018-1635-z

**Published:** 2018-03-29

**Authors:** Janette Walton, Laura O’Connor, Albert Flynn

**Affiliations:** 1 School of Food and Nutritional Sciences, University College Cork, Cork, Ireland; 2 Faculty of Health, Psychology and Social Care, Manchester Metropolitan University, C2.24 Cavendish Building, Manchester, M15 6BH UK

**Keywords:** Water, Body mass index, Waist circumference, Body fat

## Abstract

**Purpose:**

Drinking (plain) water intake has been associated with weight loss and reducing energy intake in intervention trials. In free-living populations, replacing other beverages with drinking water is associated with reduced obesity risk. However, the association of total water intake and its sources, and body fat distribution remain unevaluated. Thus, the aim of this study was to investigate total water intake and its sources and the association with anthropometric measures.

**Methods:**

Cross-sectional study of 1500 adults aged 18–90 years (Irish National Adult Nutrition Survey, 2008–2010). Total water intake and its sources were estimated using food records. Associations of total water, drinking water, beverage moisture and food moisture intakes split by tertile, and BMI (kg/m^2^), waist circumference (cm), and bio-impedance derived body fat (%) were evaluated using covariate-adjusted linear regression analyses including adjustment for energy intake and energy expenditure.

**Results:**

Higher consumption of total water was associated with lower waist circumference [*β*-coefficient (95% CI), *p* trend, tertile 3 versus tertile 1: − 2.19 (− 4.06, − 0.32), 0.036], but not BMI [− 0.44 (− 1.16, 0.28), 0.336] or body fat [− 0.87 (− 1.91, 0.17), 0.146]. Higher consumption of drinking water and food moisture were associated with lower BMI [− 0.65 (− 1.30, − 0.01), 0.027; − 0.64 (− 1.41, − 0.13), 0.014, respectively], body fat [− 1.51 (− 2.43, − 0.59), 0.001; − 1.00 (− 2.12, − 0.12), 0.001], and waist circumference [− 2.83 (− 4.51, − 1.16), < 0.001; − 1.84 (− 3.86, − 0.19), 0.082]. Beverage moisture was not associated with any of the anthropometric measurements.

**Conclusions:**

Consumption of drinking water and food moisture and not total water or beverage moisture were inversely associated with adiposity, independent of energy intake and expenditure. Advice encouraging drinking water and food moisture intake may be beneficial in addition to energy balance advice, in combating obesity.

**Electronic supplementary material:**

The online version of this article (10.1007/s00394-018-1635-z) contains supplementary material, which is available to authorized users.

## Introduction

Water is essential for life. Adequate total water intakes have been proposed [1] based on mean intakes in healthy populations. However, the role of total water intake in optimal health is not well characterised.

Low water intakes have been associated with various morbidities including urolithiasis [2], urinary tract infections, blood pressure [3], and cognitive performance [4], while total or plain water intakes have been associated with no survival advantage in a study of all-cause mortality [5]. However, evidence is piecemeal and definitive conclusions for any disease outcomes associated with low water intakes are lacking.

Research into the role of water intakes in body weight determination is more progressed. Total water intake has been positively associated with weight status in cross-sectional analyses [6, 7]. Increased drinking (plain) water intake has been associated with weight loss in trials [8–10] and, replacing other beverages, in particular sugar-sweetened beverages, with drinking water has been associated with lower risk of obesity in prospective cohorts [11, 12]. However, drinking water is not our only source of water and there is large variation in the contribution of different sources of total water intake in humans [13]. The previous investigation of dietary and body weight correlates of water intakes in the National Health and Nutrition Examination Survey (NHANES) suggests that there are differential associations of water intake and body weight and dietary characteristics depending on the dietary source of water [6]. Yet, few studies have examined total water intake and its dietary sources and adiposity.

Thus, the herein aim is to examine the cross-sectional association of total water intake, drinking water, food moisture and beverage moisture, and measured BMI, waist circumference, and body fat percentage.

## Methods

### Survey design and study population

Nationally representative data from the cross-sectional, Irish National Adult Nutrition Survey (2008–2010) were used for this analysis. 1500 free-living adults aged 18–90 years were included in the survey and all were eligible for inclusion here. Ethical approval was obtained from the Clinical Research Ethics Committee of the Cork Teaching Hospitals, University College Cork, and the Human Ethics Research Committee of University College Dublin. A detailed survey methodology is available elsewhere (http://www.iuna.net).

### Dietary intakes

All participants completed a semi-weighed 4-day food record. Daily food and beverage intakes were converted into nutrient intakes using WISP© (Tinuviel Software, Anglesey, UK) which contained McCance and Widdowson’s The Composition of Foods 6th Summary Edition and 5th Edition Supplements [14].

Total water intakes (g/day) were estimated as drinking water intake (tap and bottled), beverage moisture (water from beverages excluding drinking water), and food moisture. A detailed methodology of the estimation of water intakes has been previously published [13].

### Anthropometric measurements

Anthropometric measurements were taken in participants’ homes, during the 4 days of dietary assessment. Measurements were taken after urinary/bowel voiding, while the participant was barefoot. Height (cm) and weight (kg) were measured using standardised methods. BMI was calculated as kg/m^2^. Body fat (%) was measured using a portable Tanita body composition analyser BC-420MA (Tanita Ltd, GB). Waist circumference (cm) was measured in duplicate using a non-stretch tape measure and taken at the midpoint between the iliac crest and the bottom of the rib cage. Average waist circumference was used for analysis.

### Covariates

Socio-demographic and lifestyle data were self-reported using questionnaires. Energy expenditure was estimated using a validated questionnaire: the EPIC Physical Activity Questionnaire [15]. The alternative Mediterranean Dietary Score (aMDS), as adapted by Fung et al. [16], was calculated.

### Statistical analyses

Total water, drinking water, beverage moisture, and food moisture were each expressed as mean daily intake (g/day). Socio-demographic, anthropometric, and dietary intakes characteristics [mean ± standard deviation, median (inter-quartile range) or percent] were tabulated for the total study sample. Participants missing anthropometric data (BMI *n* = 88, body fat *n* = 181, waist circumference *n* = 223) were excluded from relevant analyses. Anthropometric measurements, energy intakes (kJ/day), and energy expenditure (MET h/week) were compared across tertile of each water intake. *p* values for trends in mean across tertile were estimated using ANOVA.

The continuous association (per 100 g/day) and categorical association (tertile) of each water intake and each anthropometric measurements was examined using multiple linear regression models. A pragmatic approach was used to account for potential confounders including demographic, lifestyle, social, dietary, and physical activity factors. Under the assumption of missing at random, missing covariate data were imputed using the sex–age-stratified mean of the total sample with available covariate data. Model 1 was adjusted for age group (18–35, 36–50, 51–64, 61–90 years, to account for non-linear association of age and BMI), sex, social class (categorical), education level (categorical), smoking status (current, past, never), alcohol consumption frequency (continuous), and season (winter, summer). Model 2 was further adjusted for the aMDS as a measure of dietary quality. Model 3 was additionally adjusted for energy expenditure (MET h/week) and energy intake (kJ/day).

Sodium, protein, and dietary fibre intakes were considered for inclusion, but sodium and protein were not significant when entered into the model, and we could not include dietary fibre due to multicollinearity with exposure variables, and therefore, none were included.

We tested for interaction between each water intake exposure and, sex, BMI (< or ≥ 25 kg/m^2^), smoking status (current or not current), energy expenditure (< or ≥ median: 86.1 MET h/week), and energy reporting status (under-reporters or accurate reporters identified using basal metabolic rate:energy intake ratio cut points [17]), in relation to anthropometric measurements. Stratified analyses were carried out where *p* ≤ 0.05. Correlation of total water, drinking water, food moisture, and beverage moisture was assessed using Pearson’s correlation coefficient. Mutual adjustment for intakes of water from other sources and using mean daily intake of total water, drinking water, food moisture, and beverage moisture were carried out as a sensitivity analyses. We also excluded participants with high intakes (top 1 percentile) and separately low intakes (bottom 1 percentile) of each of the water exposures and repeated the continuous analyses. Categorical analyses were repeated using water intake stratified by ethanol and caffeine intake (split by tertile). We examined water intakes by BMI status [underweight/normal weight (< 25 kg/m^2^), over-weight (25–30 kg/m^2^), and obese (> 30 kg/m^2^)]. *p* values for trends across BMI category were estimated using ANOVA.

Analyses were performed using Stata (version 14; Stata Corp.)

## Results

Population characteristics are displayed in Table 1. The population was representative of the Irish public for age, sex, social class, and urban/rural location when compared to Census 2006 [18]. Participants mean daily intake (men, 2504 ± 1350; women, 2079 ± 941 ml/day) of total water intake were in line with the adequate intake (men, 2500; women, 2000 ml/day) as published by the European Food Safety Authority [1]. In unadjusted analyses, energy intake was higher, and BMI, % body fat, and waist circumference were generally lower with higher water intakes (Supplementary Table 1).

**Table 1:** Socio-demographic, anthropometric, and dietary characteristics: the National Adult Nutrition Survey, Republic of Ireland (*n* = 1500)

Variable	*n* available data	Total study population
Age (year)	1500	44.5 ± 17.0^a^
Sex, men (%)	1500	49.3
Social class (%)	1500	
Professional/managerial/technical		46.7
Non-manual skilled		18.6
Manual skilled		14.8
Semi-skilled/unskilled (includes students)		19.9
Education (%)	1500	
Primary		9.4
Intermediate		20.5
Secondary		23.7
Tertiary		46.4
Energy expenditure (MET h/week)	1323	78.7 (37.3,124.0)^b^
Current smokers (%)	1480	21.0
Alcohol consumption (%)	1480	
More than once per week		60.7
Once or twice per fortnight		16.2
Infrequently or never		23
Season: Winter^c^ (%)	1500	54.3
Anthropometric measurements		
BMI (kg/m^2^)	1412	27.1 ± 5.0
Waist circumference (cm)	1277	M: 96.0 (87.0, 105.0); W: 85.0 (77.5, 94.4)
Body fat (%)	1319	M: 23.8 (18.8, 28.9); W: 34.4 (29.0, 39.6)
Water intakes	1500	
Total water (g/day)		2054 (1506, 2786)
Drinking water (g/day)		216 (0, 726)
Other beverages (g/day)		908 (557, 1350)
Food moisture (g/day)		347 (463, 870)
Dietary data	1500	
Energy (kJ)		7902 (6098, 10,335)
Alternate Mediterranean Diet Score (aMED)^d^		3.1 ± 1.6
Protein (%TE)		16.5 (13.6, 20.2)
Carbohydrate (%TE)		43.6 (37.3, 50.0)
Fat (%TE)		33.5 (27.4, 39.6)
Saturated fat (%TE)		12.8 (9.8, 16.2)
Dietary fibre (g/10 MJ)		22.1 (16.2, 29.5)
Sodium (mg/10 MJ)		2854 (2220, 3643)

*MET* metabolic equivalent tasks

^a^Mean ± SD (all such values)

^b^Median (IQR) (all such values)

^c^Winter: surveyed September–February

^d^aMED: 0–9 score, a higher overall score represents a healthier diet pattern

Comparing highest consumers (T3) to lowest consumers (T1), total water, drinking water, and food moisture intakes but not beverage moisture intakes were inversely associated with BMI after adjustment for socio-demographic covariates (model 1) and further adjustment for dietary quality (model 2) (Table 2). After further adjustment for energy balance (model 3), the associations of drinking water and food moisture, and BMI were attenuated but remained significant and the association of total water, and BMI became non-significant [*β*-coefficient (95%CI), *p* trend, T3 versus T1: − 0.44 (− 1.16, 0.28), 0.336].

**Table 2:** Cross-sectional association of total water and its main dietary sources by tertile and per 100 g/day with BMI (kg/m^2^), using multiple linear regression: the National Adult Nutrition Survey, Ireland (*n* = 1500)

	Total water				
	T1, obs = 471	T2, obs = 471	T3, obs = 470	*p* trend^1^	obs = 1412
g/day^2^	1433 (358–1834)	2171 (1835–2516)	3318 (2518–6655)		per 100 g/day
Model 1	Ref	− 0.74 (− 1.36, − 0.12)	− 0.70 (− 1.34, − 0.06)	0.052	− 0.03 (− 0.06, 0.00)
Model 2	Ref	− 0.79 (− 1.42, − 0.16)	− 0.77 (− 1.42, − 0.11)	0.037	− 0.03 (− 0.06, 0.00)
Model 3	Ref	− 0.60 (− 1.26, 0.05)	− 0.44 (− 1.16, 0.28)	0.336	− 0.01 (− 0.05, 0.01)

	Drinking water				
	T1, obs = 471	T2, obs = 471	T3, obs = 470	*p* trend^1^	obs = 1412
g/day	20 (0–113)	313 (116–554)	1144 (555–4469)		per 100 g/day
Model 1	Ref	0.04 (− 0.58, 0.65)	− 0.64 (− 1.28, − 0.00)	0.027	− 0.02 (− 0.07, 0.02)
Model 2	Ref	0.02 (− 0.60, 0.64)	− 0.69 (− 1.33, − 0.04)	0.020	− 0.03 (− 0.07, 0.02)
Model 3	Ref	0.01 (− 0.61, 0.63)	− 0.65 (− 1.30, − 0.01)	0.027	− 0.03 (− 0.07, 0.02)

	Beverage moisture				
	T1, obs = 471	T2, obs = 471	T3, obs = 470	*p* trend^1^	obs = 1412
g/day	529 (0–802)	1007 (803–1234)	1826 (1236–5281)		per 100 g/day
Model 1	Ref	− 0.42 (− 1.04, 0.20)	− 0.41 (− 1.06, 0.23)	0.230	− 0.02 (− 0.06, 0.03)
Model 2	Ref	− 0.42 (− 1.03, 0.20)	− 0.41 (− 1.05, 0.24)	0.237	− 0.02 (− 0.06, 0.03)
Model 3	Ref	− 0.26 (− 0.89, 0.37)	− 0.07 (− 0.76, 0.62)	0.897	0.01 (− 0.03, 0.06)

	Food moisture				
	T1, obs = 471	T2, obs = 471	T3, obs = 470	*p* trend^1^	obs = 1412
g/day	435 (107–563)	660 (563–772)	989 (782–1708)		per 100 g/day
Model 1	Ref	− 0.54 (− 1.15, 0.08)	− 0.77 (− 1.41, − 0.12)	0.025	− 0.12 (− 0.23, − 0.02)
Model 2	Ref	− 0.62 (− 1.25, 0.00)	− 0.96 (− 1.66, − 0.26)	0.008	− 0.16 (− 0.28, − 0.05)
Model 3	Ref	− 0.47 (− 1.11, 0.18)	− 0.64 (− 1.41, − 0.13)	0.014	− 0.11 (− 0.23, − 0.01)

Values are β-coefficients (95% confidence intervals)

Model 1 was adjusted for age group, sex, social class, education level, smoking status, alcohol consumption status, and season

Model 2 was additionally adjusted for the Alternate Mediterranean Diet Score

Model 3 was additionally adjusted for energy expenditure and energy intake

*T* tertile, *obs* observations

^1^*p*-trend was estimated by including the median value of each tertile as a continuous variable

^2^Mean (min–max), g/day

Similarly, drinking water [β-coefficient (95% CI), *p* trend, T3 versus T1: − 1.51 (− 2.43, − 0.59), 0.001], and food moisture [− 1.00 (− 2.12, − 0.12), 0.001] but not total water [− 0.87 (− 1.91, 0.17), 0.146] or beverage moisture [0.44 (− 0.56, 1.44), 0.345] were inversely associated with % body fat after adjustment for socio-demographics, dietary quality, and energy balance (Table 3). The direction of the associations was similar in continuous analyses.

**Table 3:** Cross-sectional association of total water and its main dietary sources by tertile and per 100 g/day with % body fat (%) using multiple linear regression: the National Adult Nutrition Survey, Ireland (*n* = 1500)

	Total water				
	T1, obs = 440	T2, obs = 440	T3, obs = 439	*p* trend^1^	obs = 1319
g/day^2^	1441 (358–1839)	2175 (1841–2522)	3337 (2523–6655)		per 100 g/day
Model 1	Ref	− 1.19 (− 2.09, − 0.28)	− 1.51 (− 2.44, − 0.58)	0.003	− 0.06 (− 0.10, − 0.02)
Model 2	Ref	− 1.19 (− 2.11, − 0.28)	− 1.52 (− 2.47, − 0.57)	0.003	− 0.10 (− 0.10, − 0.17)
Model 3	Ref	− 0.84 (− 1.78, 0.10)	− 0.87 (− 1.91, 0.17)	0.146	− 0.03 (− 0.07, 0.02)

	Drinking water				
	T1, obs = 457	T2, obs = 430	T3, obs = 432	*p* trend^1^	obs = 1319
g/day	20 (0–113)	335 (126–568)	116 (570–4469)		per 100 g/day
Model 1	Ref	− 0.25 (− 1.14, 0.64)	− 1.58 (− 2.50, − 0.66)	< 0.001	− 0.08 (− 0.14, − 0.01)
Model 2	Ref	− 0.25 (− 1.14, 0.64)	− 1.58 (− 2.51, − 0.65)	< 0.001	− 0.07 (− 0.14, − 0.09)
Model 3	Ref	− 0.27 (− 1.16, 0.61)	− 1.51 (− 2.43, − 0.59)	0.001	− 0.07 (− 0.13, − 0.01)

	Beverage moisture				
	T1, obs = 440	T2, obs = 440	T3, obs = 439	*p* trend^1^	obs = 1319
g/day	535 (0–807)	1007 (808–1234)	1833 (1236–5281)		per 100 g/day
Model 1	Ref	− 0.58 (− 1.47, 0.32)	− 0.30 (− 1.24, 0.63)	0.581	− 0.01 (− 0.07, 0.05)
Model 2	Ref	− 0.60 (− 1.49, 0.30)	− 0.31 (− 1.25, 0.62)	0.567	− 0.01 (− 0.07, 0.05)
Model 3	Ref	− 0.26 (− 1.17, 0.65)	0.44 (− 0.56, 1.44)	0.345	0.05 (− 0.02, 0.11)

	Food moisture				
	T1, obs = 440	T2, obs = 440	T3, obs = 439	*p* trend^1^	obs = 1319
g/day	435 (107–559)	659 (560–772)	989 (773–1708)		per 100 g/day
Model 1	Ref	− 0.74 (− 1.64, 0.15)	− 1.55 (− 2.50, − 0.60)	0.001	− 0.30 (− 0.45, − 0.15)
Model 2	Ref	− 0.80 (− 1.71, 0.11)	− 1.69 (− 2.71, − 0.66)	0.001	− 0.34 (− 0.50, − 0.17)
Model 3	Ref	− 0.48 (− 1.41, 0.46)	− 1.00 (− 2.12, − 0.12)	0.001	− 0.23 (− 0.42, − 0.05)

Values are *β* coefficients (95% confidence intervals)

Model 1 was adjusted for age group, sex, social class, education level, smoking status, alcohol consumption status, and season

Model 2 was additionally adjusted for the Alternate Mediterranean Diet Score

Model 3 was additionally adjusted for energy expenditure and energy intake

*T* tertile, *obs* observations

^1^*p*-trend was estimated by including the median value of each tertile as a continuous variable

^2^Mean (min–max), g/day

Total water, drinking water, and food moisture but not beverage moisture were inversely associated with waist circumference after adjustment for socio-demographics, dietary quality, and energy balance (Table 4). Again, the categorical (tertile) and continuous associations (100 g/day) were largely comparable.

**Table 4:** Cross-sectional association of total water and its main dietary sources by tertile and per 100 g/day with waist circumference (cm) using multiple linear regression: the National Adult Nutrition Survey, Ireland (*n* = 1500)

	Total water				
	T1, obs = 426	T2, obs = 426	T3, obs = 425	*p* trend^1^	obs = 1277
g/day^2^	1451 (373–1842)	2177 (1843–2515)	3328 (2516–6655)		per 100 g/day
Model 1	Ref	− 1.92 (− 3.55, − 0.29)	− 2.41 (− 4.08, − 0.74)	0.008	− 0.10 (− 0.17, − 0.02)
Model 2	Ref	− 2.06 (− 3.70, − 0.42)	− 2.62 (− 4.33, − 0.92)	0.004	− 0.10 (− 0.18, − 0.03)
Model 3	Ref	− 1.82 (− 3.52, − 0.11)	− 2.19 (− 4.06, − 0.32)	0.036	− 0.08 (− 0.17, − 0.01)

	Drinking water				
	T1, obs = 439	T2, obs = 416	T3, obs = 422	*p* trend^1^	obs = 1277
g/day	26 (0–125)	328 (126–563)	1147 (558–4469)		per 100 g/day
Model 1	Ref	− 0.06 (− 1.67, 1.55)	− 2.73 (− 4.38, − 1.08)	< 0.001	− 0.16 (− 0.27, − 0.05)
Model 2	Ref	− 0.12 (− 1.73, 1.49)	− 2.90 (− 4.57, − 1.23)	< 0.001	− 0.17 (− 0.29, − 0.05)
Model 3	Ref	− 0.12 (− 1.73, 1.49)	− 2.83 (− 4.51, − 1.16)	< 0.001	− 0.17 (− 0.28, − 0.05)

	Beverage moisture				
	T1, obs = 426	T2, obs = 426	T3, obs = 425	*p* trend^1^	obs = 1277
g/day	541 (0–805)	1009 (806–1242)	1831 (1242–5281)		per 100 g/day
Model 1	Ref	− 1.39 (− 3.01, 0.22)	− 0.89 (− 2.57, 0.80)	0.355	− 0.01 (− 0.12, 0.09)
Model 2	Ref	− 1.37 (− 2.99, 0.25)	− 0.87 (− 2.56, 0.81)	0.365	− 0.00 (− 0.12, 0.09)
Model 3	Ref	− 1.09 (− 2.73, 0.55)	− 0.16 (− 1.97, 1.64)	0.937	0.05 (− 0.07, 0.16)

	Food moisture				
	T1, obs = 426	T2, obs = 426	T3, obs = 425	*p* trend^1^	obs = 1277
g/day	441 (106–565)	662 (566–774)	984 (775–1708)		per 100 g/day
Model 1	Ref	− 1.16 (− 2.79, 0.47)	− 1.88 (− 3.61, − 0.15)	0.037	− 0.29 (− 0.57, − 0.01)
Model 2	Ref	− 1.38 (− 3.03, 0.27)	− 2.40 (− 4.25, − 0.54)	0.013	− 0.40 (− 0.70, − 0.09)
Model 3	Ref	− 1.12 (− 2.81, 0.57)	− 1.84 (− 3.86, − 0.19)	0.082	− 0.29 (− 0.64, − 0.05)

Values are *β*-coefficients (95% confidence intervals)

Model 1 was adjusted for age group, sex, social class, education level, smoking status, alcohol consumption status, and season

Model 2 was additionally adjusted for the Alternate Mediterranean Diet Score

Model 3 was additionally adjusted for energy expenditure and energy intake

*T* tertile, *obs* observations

^1^*p*-trend was estimated by including the median value of each tertile as a continuous variable

^2^Mean (min–max), g/day

There were no interactions for any of the outcomes with, sex (*p* = 0.061–0.805), BMI (*p* = 0.069–0.668), smoking status (*p* = 0.055–0.943), energy expenditure (*p* = 0.058–0.971), or energy reporting status (*p* = 0.336–0.732) evident.

Total water intake was positively correlated with drinking water (*r* = 0.625), beverage moisture (*r* = 0.668), and food moisture (*r* = 0.430) intakes (Supplementary Table 2). Drinking water and food moisture were also positively correlated (*r* = 0.194). Beverage moisture was not correlated with drinking water (*r* = − 0.093) or food moisture (*r* = − 0.035) intake. Mutual adjustment for intakes of water from other sources (Supplementary Table 3) did not substantially alter the associations of water intake and any of the measures of adiposity. Excluding participants with high intakes of each of the water exposures did not substantially affect the estimates, nor did stratification of water intakes to account for ethanol and caffeine intakes. Obese participants reported lower unadjusted intakes of all water intake exposures (*p* < 0.001–0.031) than their normal- or over-weight counterparts (Supplementary Table 4).

## Discussion

In summary, total water, drinking water, and food moisture intakes but not beverage moisture intake were associated with lower adiposity in socio-demographic and dietary quality adjusted analyses. Associations of drinking water and food moisture and BMI, body fat, and waist circumference were robust to adjustment for covariates including energy balance and a range of sensitivity analyses. However, adjustment for energy balance attenuated the associations of total water to non-significant.

Research into the role of beverages in the obesity epidemic has intensified in recent years [19, 20]. However, the association of water and obesity is under investigated, largely as it is not well estimated in studies. Epidemiological studies and nutritional surveys rarely assess hydration status, water intake is usually unevaluated, and fluid intake typically forms a minor part of dietary assessment. Moreover, where beverage intake has been estimated the focus is on energy and nutrient contributing beverages rather than a comprehensive assessment of fluid intake. Furthermore, drinking water, other beverages, and food moisture all contribute to total water intake but are rarely assessed together. As such, there are currently few studies to add context to our findings.

### Results in context

In contrast to our study findings, Kant et al. [6], in a cross-sectional analysis of NHANES, reported higher BMI with higher total water and beverage moisture intake and no association of drinking water and BMI. Similarly, in a more recent analysis of NHANES, Rosinger et al. [7] reported a positive association of total water intake and BMI. However, as neither analysis aimed to investigate the association of water and adiposity, these results are unadjusted for lifestyle and other dietary intakes and are likely confounded. More in keeping with our findings are the results of a cross-sectional study of 1136 Japanese female students [21]. In analyses adjusted for lifestyle factors including physical activity, energy expenditure, and energy intake, the authors report an inverse association of food moisture, and BMI and waist circumference but no association of beverage moisture, and BMI or waist circumference. There were methodological differences between this study and our herein presented work. In this study, drinking water was included as beverage moisture, total water intake was not estimated, and the authors did not adjust for dietary quality.

The previous intervention trials have shown that increasing drinking (plain) water intake can promote weight loss [8–10]. This is reportedly through reducing caloric intake and not through the consumption of water per se. In epidemiological studies, replacement of drinking water intake with other beverages, in particular sugar-sweetened beverages, has been associated with risk of obesity [11, 12]. Therefore, it could be argued that the inverse associations of drinking water with adiposity observed in this study are a replacement effect. However, mutual adjustment for other water sources in this study did not substantially change the interpretation of our results, suggesting a direct effect.

Beverage moisture intake was not associated with adiposity in this study. Considering intake of sugar-sweetened beverages has been shown to be associated with adiposity independent of caloric contribution [22, 23], but no causal agent or mechanism has been confirmed, it is possible that this null association is due to residual confounding. The heterogeneity of the beverage moisture group may also explain the null association as contribution of individual food and beverage types to water intakes may have moderated effects, and the inclusion of diet beverages may have led to reverse causality as participants may have preferentially consumed diet beverages in trying to lose weight.

### Mechanisms

The mechanisms in which water may affect adiposity are unknown. Water intake may affect adiposity in part by reducing energy intake through effects on satiety [9], but it fails to explain the associations reported here as the reported inverse associations are adjusted for energy intake and expenditure. Furthermore, there is the possibility of a synergistic effect of water and another dietary component potentially water and dietary fibre intake. This is a potential mechanism worth considering given the requirement of adequate hydration for optimal functioning of soluble fibre. It is also possible that there is water induced thermogenesis and/or fat oxidation. This has been examined in intervention trials for drinking water intake, but there is little consensus. Boshmann et al. [24, 25] first proposed the mechanism that, however, it has been recently refuted [26]. However, none of these trials controlled for total water intake and further investigation is needed.

### Strengths and limitations

There are a number of strengths to this research. The National Adult Nutrition Survey is a well-characterised study with objectively measured anthropometrics. This allows for adjustment of a wide-range of potential confounders, including intakes of diuretics (ethanol and caffeine) and the aMDS as a proxy of overall dietary quality. The main strength of these analyses is the availability of detailed water intake data that are not usually collected in dietary intake studies. The limitations of this research are also worth consideration. This is a cross-sectional study and thus cannot inform causality or the direction of the association. However, as the investigation of the water–adiposity association is in its infancy, these analyses offer worthwhile information. Dietary intakes were self-reported albeit from prospective semi-weighed food records. As such, we cannot rule out reporting bias. Selective reporting bias by BMI level may also be present. However, in this study, those who were over-weight and obese reported lower intakes of water exposures when compared with their normal weight counterparts. Therefore, any selective reporting bias present in this study would mean that our results are biased towards the null and are underestimates. Finally, as with all observational studies, we cannot rule out residual confounding or potential for endogeneity, which may partially or wholly explain the findings.

These results may be generalisable to other similar adult populations. However, when evaluating generalisability, it should be considered that the sample is largely white Irish and lives in a temperate oceanic climate and thus may have lower range of water intakes than hotter countries. Furthermore, minerals and contaminates in water are not adjusted for in these analyses and may vary geographically potentially changing the dietary exposure to a component causal in the association.

## Conclusion

Consumption of drinking water and food moisture may be inversely associated with adiposity, independent of energy intake and expenditure. Public health initiatives and dietary advice encouraging drinking water and food moisture intake may be beneficial in addition to energy balance advice in combating the obesity epidemic.

## Electronic supplementary material

Below is the link to the electronic supplementary material.

Supplementary material 1 (XLSX 26 KB)
